# Consumption of *Bt* Rice Pollen Containing Cry1C or Cry2A Protein Poses a Low to Negligible Risk to the Silkworm *Bombyx mori* (Lepidoptera: Bombyxidae)

**DOI:** 10.1371/journal.pone.0102302

**Published:** 2014-07-11

**Authors:** Yan Yang, Yue Liu, Fengqin Cao, Xiuping Chen, Lisheng Cheng, Jörg Romeis, Yunhe Li, Yufa Peng

**Affiliations:** 1 College of Environment and Plant Protection, Hainan University, Haikou, China; 2 State Key Laboratory for Biology of Plant Diseases and Insect Pests, Institute of Plant Protection, Chinese Academy of Agricultural Sciences, Beijing, China; 3 Qiongtai Teachers College, Haikou, China; 4 Agroscope, Institute for Sustainability Science ISS, Zurich, Switzerland; University of Tennessee, United States of America

## Abstract

By consuming mulberry leaves covered with pollen from nearby genetically engineered, insect-resistant rice lines producing Cry proteins derived from *Bacillus thuringiensis* (*Bt*), larvae of the domestic silkworm, *Bombyx mori* (Linnaeus) (Lepidoptera: Bombyxidae), could be exposed to insecticidal proteins. Laboratory experiments were conducted to assess the potential effects of Cry1C- or Cry2A-producing transgenic rice (T1C-19, T2A-1) pollen on *B. mori* fitness. In a short-term assay, *B. mori* larvae were fed mulberry leaves covered with different densities of pollen from *Bt* rice lines or their corresponding near isoline (control) for the first 3 d and then were fed mulberry leaves without pollen. No effect was detected on any life table parameter, even at 1800 pollen grains/cm^2^ leaf, which is much higher than the mean natural density of rice pollen on leaves of mulberry trees near paddy fields. In a long-term assay, the larvae were fed *Bt* and control pollen in the same way but for their entire larval stage (approximately 27 d). *Bt* pollen densities ≥150 grains/cm^2^ leaf reduced 14-d larval weight, increased larval development time, and reduced adult eclosion rate. ELISA analyses showed that 72.6% of the Cry protein was still detected in the pollen grains excreted with the feces. The low exposure of silkworm larvae to Cry proteins when feeding *Bt* rice pollen may be the explanation for the relatively low toxicity detected in the current study. Although the results demonstrate that *B. mori* larvae are sensitive to Cry1C and Cry2A proteins, the exposure levels that harmed the larvae in the current study are far greater than natural exposure levels. We therefore conclude that consumption of *Bt* rice pollen will pose a low to negligible risk to *B. mori*.

## Introduction

Rice, *Oryza sativa* L., is a staple food for more than half of the world's population and for over 65% of the Chinese people [1], [2]. To feed a growing population worldwide, rice production will have to increase by more than 40% by the year 2030 [3]. Similarly, China will need to increase its rice production by at least 20% by 2030 in order to meet its domestic needs [4]. Rice production, however, is constrained by many factors, and insect pests are among the most important [5].

Insect pests that can substantially reduce rice production in China include the following lepidopteran species. Recent research has confirmed that genetic engineering of rice is an efficient strategy for insect pest control. Multiple insect-resistant genetically engineered (IRGE) rice lines have been developed that produce Cry toxins derived from the bacterium *Bacillus thuringiensis* (*Bt*), and these IRGE rice lines are very effective against these lepidopteran pests [5]–[7].

Before a novel GE variety is commercialized, its potential risks to the environment and animal and human health must be extensively evaluated, and an important component of the risk assessment concerns the potential effects of IRGE crops on non-target organisms [8], [9]. Many laboratory and field studies have demonstrated that *Bt* rice represents a negligible threat to non-target arthropods belonging to orders that differ from that of the target pests, i.e., *Bt* proteins produced in current IRGE rice lines only affect lepidopterans [5], [9]–[14]. On the other hand, non-target lepidopterans could be affected by *Bt* rice and therefore warrant special attention in the risk assessment of IRGE crops [15]. A non-target lepidopteran of particular concern in China is the silkworm *Bombyx mori* Linnaeus (Lepidoptera: Bombycidae).

*B. mori* is an economically and culturally important insect in China, which is a world center of silk production [16]. *B. mori* larvae feed exclusively on mulberry (*Morus atropurpurea* Roxb.) leaves. In southeast China, mulberry trees are typically planted near or around rice fields in a planting system that is referred to as mulberry-mixed cropping [17]. Thus, once *Bt* rice is commercially grown in China, mulberry leaves may be covered with *Bt* rice pollen. It follows that *B. mori* larvae could be exposed to Cry proteins if they consume mulberry leaves covered with *Bt* rice pollen and if Cry proteins are produced in the pollen [18]–[22]. Because *B. mori* belongs to the same order as the target pests, i.e., the Lepidoptera, it may be sensitive to lepidopteran-active Cry proteins produced by the current *Bt* rice lines. Thus, before *Bt* rice lines are approved for commercial use, their potential effects on *B. mori* should be assessed [5].

In the current study, we developed and used a rice pollen-feeding assay to assess the potential effects of *Bt* rice pollen containing Cry2A or Cry1C protein on *B. mori* larvae.

## Results

### *Bt* protein contents in rice pollen

No *Bt* protein was detected in pollen from the control rice (Minghui 63). The mean (±SE) content of Cry2A was 28.15±1.19 µg/g dry weight (DW) in T2A-1 pollen, which was more than 11-fold higher than the content of Cry1C in T1C-19 pollen (2.40±0.08 µg/g DW).

### Pollen consumption by *B. mori*

**Figure 1:**
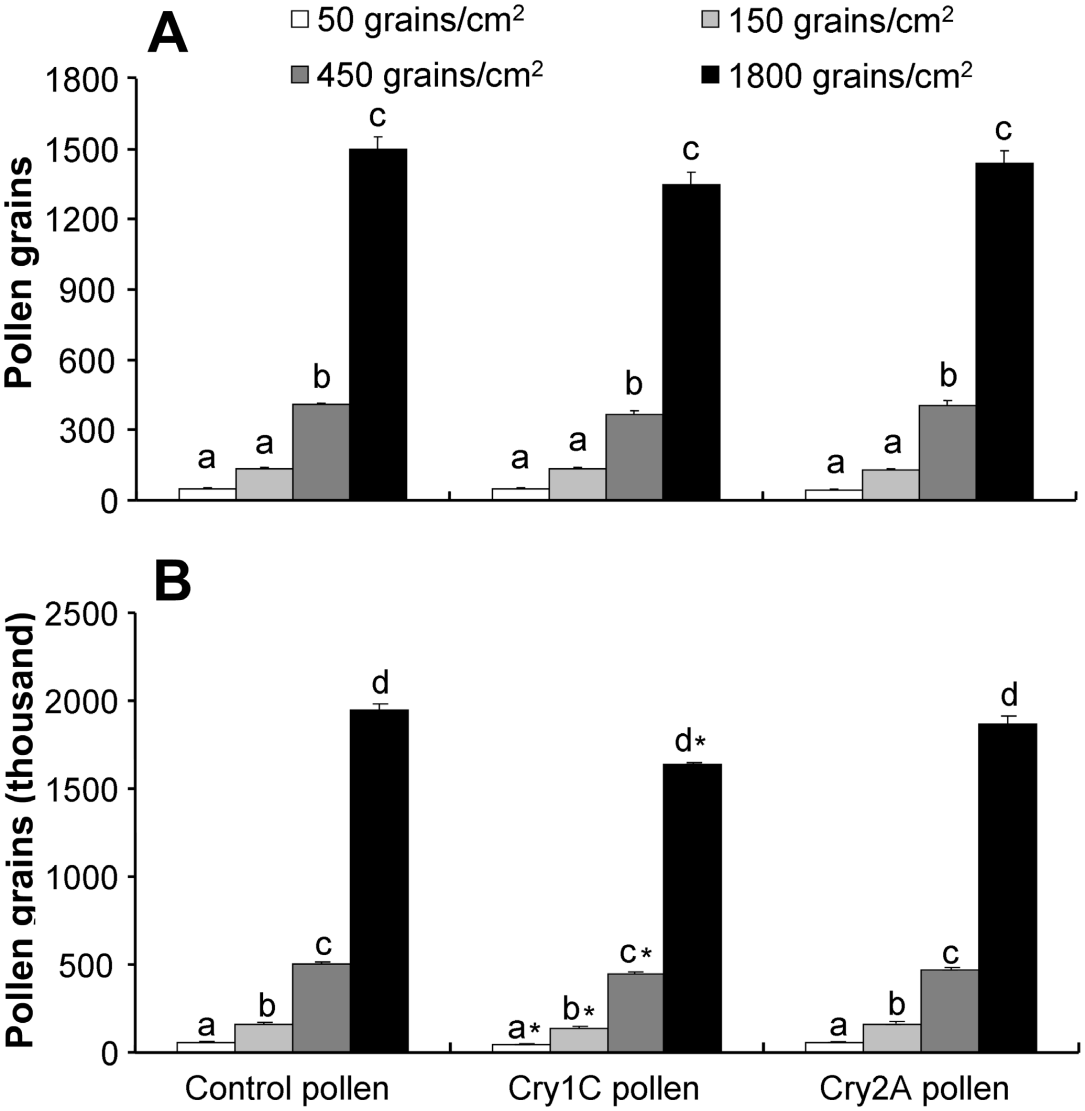
Rice pollen consumption per *Bombyx mori* larva. Lavae were fed mulberry leaves covered with different doses of pollen from Cry1C- or Cry2A-expressing *Bt* rice or pollen from the corresponding non-transformed varieties for (A) 3 d (short-term assay) and for (B) the entire larval stage (long-term assay). For each pollen type within each assay, bars with different letters are significantly different (one-way ANOVA with Tukey test), while asterisks indicate significant differences between the *Bt* pollen treatment and the corresponding non-*Bt* pollen treatment at the same pollen dose (Dunnett test). Values are means + SE, n = 3.

Pollen consumption per larva increased as the density of pollen on the mulberry leaf squares increased (one-way ANOVA; *P*<0.01 for each type of rice pollen). Pair-wise comparisons by Tukey HSD tests showed significant differences between any two pollen doses in both bioassays except for the two lowest doses in the short-term bioassay (all *P*<0.001) (Fig. 1A&B).

In the short-term bioassay, the number of pollen grains consumed was independent of pollen type (Cry1C, Cry2A, and control rice pollen) (Dunnett test; all *P*>0.1 at any pollen density) (Fig. 1A). In the long-term assay, however, consumption was lower with Cry1C pollen than with control pollen (*P*<0.05 for any pollen density) but did not differ (*P*>0.05 for any pollen density) between Cry2A pollen and control pollen (*P*>0.05) (Fig. 1B).

### Effects of pollen consumption on larval weight

In both bioassays, 14-d larval weight was not affected by increased pollen consumption regardless of the source of the pollen (one-way ANOVA for the short-term assay; control: *F* = 0.36, df = 17, *P* = 0.832; Cry1C: *F* = 0.16, df = 17, *P* = 0.96; Cry2A: *F* = 0.33, df = 17, *P* = 0.86; one-way ANOVA for the long-term assay; control: *F* = 0.20, df = 17, *P* = 0.93; Cry1C: *F* = 2.79, df = 17, *P* = 0.07; Cry2A: *F* = 1.96, df = 17, *P* = 0.16). In the short-term assay at any pollen density, 14-day larval weight did not differ significantly between either *Bt* treatment and the control (Dunnett's test; *P*>0.1). In the long-term assay, however, larval weight was significantly lower with *Bt* rice pollen than with control pollen at the pollen dose of 150 (Cry1C: *P* = 0.015; Cry2A: *P* = 0.018), 450 (Cry1C: *P* = 0.015; Cry2A: *P* = 0.012), and 1800 grains/cm^2^ leaf (Cry1C: *P* = 0.033; Cry2A: *P* = 0.033) (Table 1).

**Table 1:** Life table parameters of *Bombyx mori* larvae when fed pollen from Cry1C- or Cry2A-expressing *Bt* rice or pollen from the corresponding non-transformed varieties.

Parameter	No. of pollen grains/cm^2^ leaf	Short-term assay^1^			Long-term assay^2^		
		Control pollen	Cry1C pollen	Cry2A pollen	Control pollen	Cry1C pollen	Cry2A pollen
Larval weight (mg)	0	400.91±14.70 a	400.91±14.70 a	400.91±14.70 a	400.91±14.70 a	400.91±14.70 a	400.91±14.70 a
	50	394.98±13.42 a	399.64±17.07 a	389.49±22.92 a	404.18±12.18 a	380.03±5.31 a	384.13±22.84 a
	150	410.48±10.80 a	398.88±4.62 a	383.40±10.56 a	415.31±9.41 a	366.86±8.41 a*	371.34±6.98 a*
	450	396.30±12.12 a	388.54±23.64 a	375.96±25.52 a	410.00 ±11.49 a	356.02±2.64 a*	359.17±5.18 a*
	1800	385.45±4.09 a	386.24±18.18 a	388.89±4.21 a	412.40±8.82 a	347.25±17.51 a*	354.32±4.69 a*
Larval developmental time (d)	0	26.99±0.37 a	26.99±0.37 a	26.99±0.37 a	26.99±0.37 a	26.99±0.37 a	26.99±0.37 a
	50	26.67±0.49 a	27.00±0.21 a	27.41±0.16 a	27.33±0.24 a	27.70±0.12 a	27.27±0.12 a
	150	26.97±0.28 a	27.57±0.20 a	27.67±0.15 a	27.47±0.19 a	27.83±0.09 a	28.07±0.30 b
	450	27.60±0.12 a	27.83±0.13 a	27.27±0.19 a	27.57±0.24 a	28.90±0.15 b*	28.37±0.15 b
	1800	27.30±0.15 a	27.53±0.07 a	27.45±0.23 a	27.90±0.40 a	29.63±0.15 b*	29.17±0.35 b
Pupation rate (%)	0	86.67±3.57 a	86.67±3.57 a	86.67±3.57 a	86.67±3.57 a	86.67±3.57 a	86.67±3.57 a
	50	90.00±0.00 a	85.00±2.89 a	81.67±1.67 a	80.00±2.89 a	80.00±2.89 ab	85.00±2.89 a
	150	83.33±1.67 a	83.33±4.41 a	83.33±1.67 a	81.67±1.67 a	73.33±1.67 ab	71.67±3.33 ab
	450	78.33±3.33 a	80.00±5.00 a	81.67±6.01 a	76.67±1.67 a	73.33±1.67 ab	75.00±2.89 ab
	1800	80.00±0.00 a	81.67±3.33 a	70.00±2.89 a	71.67±3.33 a	66.67±1.67 b	65.00±2.89 b
Eclosion rate (%)	0	73.33±2.47 a	73.33±2.47 a	73.33±2.47 a	73.33±2.47 a	73.33±2.47 a	73.33±2.47 a
	50	48.33±1.67 b	53.33±1.67 b	48.33±4.41 b	45.00±2.89 b	38.33±6.01 b	41.67±4.41 b
	150	41.67±4.41 b	35.00±2.89 c	28.33±3.33 c	31.67±4.41 bc	18.33±6.01 c	28.33±4.41 bc
	450	41.67±6.01 b	30.00±5.00 c	31.67±4.41 c	30.00±2.89 bc	6.67±1.67 c*	25.00±2.89 c
	1800	33.33±1.67 b	30.00±2.89 c	-	23.33±6.01 c	1.67±1.67 c*	3.33±1.67 d*

Values are means ± SE, n = 3.

1 Neonates of *B. mori* were fed mulberry leaves covered with rice pollen for the first 3 d and were then fed mulberry leaves without rice pollen until pupation.

2 Neonates of *B. mori* were fed mulberry leaves covered with rice pollen for their entire larval stage.

“-” denotes lost data.

For each parameter, means in a column followed by different letters are significantly different (one-way ANOVAs with Tukey tests for larval weight, pupation and eclosion rate; Kruskal-Wallis Tests followed by Mann-Whitney U-Tests for larval development time).

An asterisk denotes a significant difference between the *Bt* pollen treatment and the corresponding non-*Bt* pollen treatment at the same pollen dose (Dunnett test).

### Effects of pollen consumption on larval development

Larval developmental time was not significantly affected by the increased pollen consumption of any rice pollen in the short-term assay (Kruskal-Wallis test; control: *χ^2^* = 4.77, *P* = 0.31; Cry1C: *χ^2^* = 7.88, *P* = 0.10; Cry2A: *χ^2^* = 2.94, *P* = 0.57) (Table 1). In the long-term assay, developmental time was not affected by an increase in the density of control pollen (*χ^2^* = 3.63, *P* = 0.46) but was significantly increased by an increase in the density of both kinds of *Bt* pollen (*χ^2^* = 14.378, *P* = 0.006 for Cry1C, and *χ^2^* = 13.769, *P* = 0.008 for Cry2A) (Table 1). In the short-term assay, pollen type did not significantly affect larval developmental time (Dunnett's test, all *P*>0.10). In the long-term assay, however, larval developmental time was significantly longer with Cry1C pollen than with control pollen at doses of 450 and 1800 grains/cm^2^ (both *P*<0.05). Larval developmental time did not differ between Cry2A and control pollen at any pollen density (all *P*>0.05) (Table 1).

### Effects of pollen consumption on pupation and eclosion rate

In the short-term assay, the pupation rate was not significantly affected by pollen density on the mulberry leaves (one-way ANOVA; control: *F* = 2.06, df = 17, *P* = 0.15; Cry1C: *F* = 0.47, df = 17, *P* = 0.76; Cry2A: *F* = 2.70, df = 17, *P* = 0.08) (Table 1), although the pupation rate tended to drop with the highest density of Cry2A pollen. In the long-term assay, the pupation rate gradually decreased with the increase of pollen density; the decrease was marginally significant with control pollen (*F* = 3.12, df = 17, *P* = 0.05), but was significant with both Cry1C pollen (*F* = 6.58, df = 17, *P* = 0.004) and Cry2A pollen (*F* = 6.77, df = 17, *P* = 0.004).

In both bioassays, eclosion rate of the silkworm significantly decreased as pollen density increased (one-way ANOVA; *P*<0.001 for any pollen type). In the short-term assay, the eclosion rate was similar with *Bt* pollen and control pollen (Dunnett's test; *P*>0.2 for any pollen density). In the long-term assay, however, the eclosion rate was significantly lower with Cry1C pollen than with control pollen at 450 and 1800 grains/cm^2^ leaf (*P* = 0.001 and 0.011, respectively) or with Cry2A pollen than with control pollen at 1800 grains/cm^2^ leaf (*P* = 0.016) (Table 1).

### Fate of Cry1C contained in *Bt* rice pollen after silkworm gut passage

Pollen grains from *Bt* rice T1C-19 contained 27.4% less Cry1C protein after passage through the digestive system of *B. mori* larvae. This difference, however, was not statistically significant (Student's-*t* test, *t* = 1.96, df = 4, *P* = 0.12) (Table 2). Observations under the microscope revealed that the majority of the pollen grains were only partly digested.

**Table 2:** Cry1C protein content of rice pollen grains before and after passage through the digestive system of *Bombyx mori* larvae.

Sample	Cry1C concentration (**µ**g/g dry weight)	No. of pollen grains per mg	Cry1C content per grain (pg) (*c*_x_ = *a*_x_/*b*_x_×10^3^)	Lost rate of Cry1C protein (%) (*R*_Cry_ = (*c*_1_-*c*_2_)/*c*_1_×100)
Fresh pollen	3.62±0.10 (*a*_1_)	43106.62±1140.13 (*b*_1_)	0.084±0.002 (*c*_1_)	27.40
Feces	0.08±0.03 (*a*_2_)	1026.01±68.66 (*b*_2_)	0.061±0.011 (*c*_2_)	

Insects were fed with mulberry leaves covered with T1C-19 rice pollen, and the feces were collected; n = 3.

Values are means ± SE in columns 2 and 4.

## Discussion

The risk represented by a *Bt* crop for a non-target organism depends on the organism's sensitivity to the *Bt* protein and on the probability that it is exposed to harmful concentrations of that protein in the field [8], [23]. Therefore, when dietary assays are used to assess the effects of *Bt* pollen on a non-target species, selection of appropriate pollen doses is important and should be based on the pollen densities that the species may encounter in the field [24]. Fan et al. [17] reported that the density of rice pollen deposited on the leaves of mulberry trees near rice fields ranged from 13–199 grains/cm^2^ with an average density of 93 grains/cm^2^. Yao et al. [20] reported that the maximum density of rice pollen on mulberry leaves was 1636 grains/cm^2^, although the probability of that density occurring in the field was only 0.2%. In the current study, the pollen doses tested ranged from 0 to 1800 grains/cm^2^ of mulberry leaf, which covered the potential pollen densities to which silkworms may be exposed in the field. In addition, both a short-term feeding assay (3 d) and a long-term feeding assay (27 d, covering the entire larval stage) were conducted. In the field, rice anthesis usually lasts 10 to 15 d [20], [21] and pollen is typically shed at a high rate for less than 1 week (Yunhe Li et al., unpublished data). In addition, the silkworm larval period may not totally overlap with rice anthesis, and environmental factors such as rain and wind will also affect rice pollen deposition on mulberry leaves. Therefore the short-term assay may represent an realistic exposure scenario and the long-term assay represents a worst-case scenario. Thus, the assays used in this study are useful for assessing the risk of that *Bt* rice pollen represents to the silkworm, *B. mori*.

As expected, the number of pollen grains consumed by *B. mori* larvae increased as the density of rice pollen on mulberry leaves increased. Interestingly, the larvae in the long-term assay consumed significantly less Cry1C pollen than control pollen even at the low dose of 50 grains/cm^2^ and even though this density of Cry1C pollen did not affect survival or development. This suggests that the reduced consumption of Cry1C pollen was not caused by harm to the larvae and that Cry1C protein may have antifeedant activity towards *B. mori* larvae. This was not the case with Cry2A pollen in the long-term assay in that the larvae consumed similar quantities of Cry2A and control pollen.

In both bioassays, the larval weight, development time, and pupation rate were not negatively affected by consumption of control rice pollen even at the highest pollen dose. It seems that consumption of control rice pollen does not affect the normal development of *B. mori* larvae. Effects seen in the *Bt* pollen treatments can thus be attributed to the *Bt* Cry toxins. Surprisingly, however, the eclosion rate of larvae was significantly decreased by consumption of control or *Bt* pollen even at the lowest dose of 50 grains/cm^2^ leaf in both bioassays. The biological mechanism underlying this effect is unclear. These results suggest that dietary effects can be specific to certain life table parameters. It follows that as many parameters as possible should be observed in such dietary bioassays.

No adverse effect was detected for *Bt* pollen in the short-term assay, even at the highest pollen density of 1800 grains/cm^2^ leaf. In the long-term assay, however, Cry1C or Cry2A pollen negatively affected all of the tested *B. mori* life table parameters with an exception of pupation rate. Even at a dose of only 150 grains/cm^2^ leaf, *Bt* pollen significantly reduced larval weight. This is consistent with Wang et al. [18], who reported that *B. mori* larval weight but not survival was reduced when the larvae were fed *Bt* rice pollen containing Cry1Ab toxin. Although we did not statistically compare Cry1C pollen and Cry2A pollen treatments, the data indicate that consumption of Cry1C pollen was more harmful than consumption of Cry2A pollen. For example, *B. mori* larval developmental time was significantly increased by feeding on Cry1C pollen at 450 and 1800 grains/cm^2^ leaf but was not increased by feeding on Cry2A pollen even at 1800 grains/cm^2^ leaf. Our ELISA determination indicated that the Cry2A content in T2A-1 rice pollen was 11-times higher than the Cry1C content in T1C-19 rice pollen. This shows that Cry1C is much more toxic than Cry2A to *B. mori* larvae, which is also the case for other lepidopterans including the stem borer *C. suppressalis*, a target pest of the *Bt* rice lines. In sensitive-insect bioassays, neonate larvae of *C. suppressalis* were fed for 7 d with artificial diet containing a range of Cry protein concentrations. The EC_50_ (toxin concentration resulting in 50% weight reduction compared to the control) was 18 ng/mL diet for Cry1C [25] and 1310 ng/mL diet for Cry2A [12]. Previous research has demonstrated that different Cry proteins can have significantly different insecticidal spectra even if they have high homology. For example, Cry1Aa, Cry1Ab, and Cry1Ac have similar structures and belong to the same taxonomic class [26]. In assays with *B. mori*, however, Cry1Aa was 17-times more toxic than Cry1Ab [27] and 400-times more toxic than Cry1Ac [28]. Based on the EC_50_ values reported for Cry1C and Cry2A, we would have expected a higher mortality in the silkworm larvae fed with high doses of *Bt* rice pollen. A likely explanation for the relatively low toxicity is that the rice pollen grains were only partly digested in the larval gut and thus the larvae were only exposed to a fraction of the Cry protein. This was confirmed by the fact that 72.6% of the Cry protein was detected in the pollen grains excreted with the feces.

That *cry1C*- and *cry2A*-expressing *Bt* rice pollen are toxic to the lepidopteran *B. mori* is not surprising because Cry1 and Cry2 proteins are specifically toxic to lepidopterans. Our results do not indicate, however, that the growing of these *Bt* rice lines will pose a significant risk to the silk industry for the following reasons. First and as discussed earlier, our long-term pollen exposure assay represents a worst-case scenario, and *B. mori* larvae are unlikely to be exposed to rice pollen for such a long period [20], [21], [29]. Second, the negative effects were detected only at the relatively high pollen densities of ≥150 grains/cm^2^ mulberry leaf, which rarely occur in the field. Yao et al. [20] reported a mean of 62.3 grains/cm^2^ of mulberry leaf at a distance of 0 m from the paddy field edge, and the density steeply declined to 4.0 grains/cm^2^ of leaf at a distance of 10 m. Third, the mulberry leaves that are fed to *B. mori* larvae are picked from trees and transferred to a building where the larvae are fed. Thus, the quantity of rice pollen deposited on the leaves would likely be reduced during transport and handling [20]. Fourth, the *Bt* rice pollen used in our assays was fresh and protected from sunlight and other environmental factors, which would not be the case for *Bt* rice pollen in the field; in the field, the activity of the *Bt* proteins in rice pollen may be partially reduced by exposure to rainfall and sunlight before the mulberry leaves are picked and fed to the larvae [20], [30]. Considering all four reasons and the results of our short-term bioassay, we conclude that the impact of T1C-19 and T2A-1 rice pollen on the silkworm, *B. mori*, is probably minimal.

Although exposure of *B. mori* larvae to *Bt* rice pollen is likely to be limited under natural conditions, *B. mori* has received substantial attention in the risk assessment of *Bt* rice because of its economic importance [18]–[22], [31]. Wang et al. [18] found that consumption of *Bt* rice pollen containing Cry1Ab protein at a density of 110 grains/cm^2^ mulberry leaves during the whole larval development did not affect the survival of the silkworms, but significantly reduced larval weight. Yao et al. [20] reported that *Bt* rice pollen containing a fusion Cry1Ab/Ac protein had no negative effect on *B. mori* larvae, when their neonates were exposed to *Bt* pollen at the density of 3395 grains/cm^2^ mulberry leaves for 48 h. Likewise, two subsequent studies did not find detrimental effects of Cry1Ab-containing rice pollen from *Bt* rice lines KMD1 and B1 on *B. mori* larvae [19], [21]. That different studies have reported differences in the toxicity of *Bt* pollen containing Lepidoptera-active Cry proteins to *B. mori* larvae can be explained as follows: i) the studies used rice pollen that contained different types of *Bt* proteins; ii) the concentrations of *Bt* proteins in the rice pollen differed; and especially iii) the *Bt* protein exposure differed among studies [21]. Although *Bt* rice pollen was found to be toxic to *B. mori* larvae in some studies, previous researchers have generally concluded that *Bt* rice pollen poses a negligible risk to this domesticated lepidopteran [5], [21].

Ours is the first study to assess the effects of Cry1C- and Cry2A-containing rice pollen on *B. mori* larvae. Although the results indicate that the larvae are sensitive to Cry1C and Cry2A proteins contained in T1C-19 and T2A-1 rice pollen, the results also indicate that the *Bt* rice lines probably represent a low to negligible risk to *B. mori* larvae because of the limited exposure of the larvae to *Bt* rice pollen under natural conditions. To guarantee the safety of the silk industry, however, we recommend that mulberry leaves on trees that are near paddy fields planted with *Bt* rice lines should not be used to feed *B. mori* larvae or should be washed before they are fed to *B. mori* larvae.

## Materials and Methods

### Ethics statement

No specific permits were required for the described field studies. The rice fields from which rice pollen were collected were owned by the author's institute (Institute of Plant Protection, Chinese Academy of Agricultural Sciences, CAAS). These field studies did not involve endangered or protected species.

### Insects

A hybrid of *B. mori*, Liangguang 1, was used in this study. Eggs of *B. mori* were purchased from the Hainan Silk Development Co., Ltd. (Qiongzhong County, Hainan, China) and were kept in a climatic chamber at 27±0.5°C, 75±5% RH, and 12:12 h L:D photoperiod. Newly hatched larvae (<12 h after hatching) were used for all experiments.

### Plant materials

Two transgenic rice varieties, T1C-19 and T2A-1, and their corresponding non-transformed near isoline Minghui 63 were used for the experiments. T2A-1 plants express a synthesized modified *cry2A* gene and T1C-19 plants express a modified *cry1C* gene targeting lepidopteran rice pests. Minghui 63 is an elite indica restorer line for cytoplasmic male sterility in China. All rice seeds were kindly provided by Prof. Yongjun Lin (Huazhong Agricultural University, Wuhan).

The rice lines were simultaneously planted in three adjacent plots at the experimental field station of the Institute of Plant Protection, CAAS, near Langfang city, Hebei Province, China (39.5°N, 116.4°E). Each plot was approximately 0.1 hectare, and plots were separated by a 1-m ridge. The rice seeds were sown in a seeding bed on 6 May 2012, and the seedlings were transplanted to the experimental plots on 14 June 2012 when the seedlings were at the four-leaf stage. The plants were cultivated according to the common local agricultural practices but without pesticide sprays.

During rice anthesis from 3 to 13 September 2012, rice pollen was collected daily by shaking the rice tassels in a plastic bag. The collected pollen was air dried at room temperature for 48 h and subsequently passed through a screen with 0.125-mm openings to remove anthers and contaminants. Pollen collected from each rice line was pooled and stored at −80°C until used.

The leaves of mulberry were collected from a mulberry garden at Qiongzhong County, Hainan Province, China. The freshly collected mulberry leaves were washed in water, air dried at room temperature, and stored at 4°C. The leaves were used within 4 days of collection.

### *Bt* protein content in rice pollen

The concentrations of Cry1C and Cry2A proteins in pollen were measured with double-antibody sandwich enzyme-linked immunosorbent assay (DAS-ELISA) kits from EnviroLogix Inc. (Portland, ME, USA; Catalog No. AP 007and kit lot 140433N for Cry1C, and AP 005 and 202482 for Cry2A). Five samples (5–10 mg) of *Bt* or control rice pollen were lyophilized and then homogenized in 1 ml of PBST with a micro-mortar and pestle on ice. After centrifugation and appropriate dilution of the supernatants, ELISA was performed according to the manufacturer's instructions. The optical density (OD) values were read with a microplate spectrophotometer (PowerWave XS2, BioTek, USA). The concentrations of Cry1C and Cry2A were calculated by calibrating the OD values to a range of concentrations of standard Cry1C and Cry2Aa samples provided with the kit.

### Bioassays

Two bioassays were conducted in a climate chamber at 27±0.5°C, 75±5% RH, and a 12:12 h L:D photoperiod. In a short-term assay, neonates of *B. mori* were fed cut mulberry leaves (described later in this paragraph) that were covered with rice pollen for the first 3 d and then were fed with cut mulberry leaves without rice pollen for the remainder of the larval period. In a long-term assay, *B. mori* were fed cut mulberry leaves with rice pollen for their entire larval period. For both assays, a scissors was used to cut mulberry leaves into squares of different sizes. Squares that were 1, 4, 30, and 50 cm^2^ were used for feeding the first, second, third, and fourth and fifth instar larvae, respectively. Each bioassay included three main treatments: mulberry leaf squares with control rice pollen, Cry1C pollen, or Cry2A pollen. Each main treatment included five doses of rice pollen so that each cm^2^ of mulberry leaf square contained 0 or about 50, 150, 450, or 1800 pollen grains.

To obtain the different pollen doses on the mulberry leaf squares, the mean weight of a single rice pollen grain was estimated using the method described in Li et al. [32]. Based on the individual weight of a rice pollen grain, the appropriate quantity of rice pollen grains was weighed and placed in a plastic Petri dish. The Petri dish was shaken by hand after a single wet leaf square was placed in the dish. Examination of leaf squares with a stereo-microscope (50×) confirmed that the actual densities of pollen grains that adhered to the leaf surfaces were very similar to the expected doses, and that the pollen grains were relatively evenly distributed.

Plastic boxes (12×7×6 cm for 1^st^ to 3^rd^ instar larvae and 35×25×20 for 4^th^ and 5^th^ instar larvae) with small holes in the lids were used for both feeding assays. A single treated leaf square was placed on a filter paper on the bottom of a box, and 20 randomly selected *B. mori* neonates were placed on the leaf square. Three replicates and a total of 60 insects were tested for each pollen dose. The number of alive larvae was recorded daily. When a leaf square was almost completely consumed, it was replaced with a new pollen-treated or untreated leaf square. When leaf squares were changed, the uneaten leaf area was calculated using the method of Lang and Vojtech [33]; this information was needed to estimate the mean amount of pollen grains consumed by each larva. When larvae developed into 5^th^ instars and stopped eating, a net was introduced into the plastic dish for larval cocooning. The assays were terminated when all of the insects had developed into adults or died. The following variables were determined: pupation rate, eclosion rate, larval development time, and 14-day larval weight.

### Fate of Cry1C contained in *Bt* rice pollen after silkworm gut passage

To estimate the degree at which silkworm larvae are exposed to Cry protein when *Bt* rice pollen grains pass through their gut, the mean Cry1C content in pollen grains before ingestion or from pollen grains in the feces of the silkworm larvae was compared. Neonates of *B. mori* were fed mulberry leaves until the third instar and then starved for 24 h to empty their gut. Subsequently, the larvae were placed in 3 plastic boxes (10 insects per box) and provided with mulberry leaves covered with *Bt* rice pollen of T1C-19 at a density of >1800 grains/cm^2^. After 12 h, the silkworm larvae were transferred to new boxes and received the same food. Subsequently, fresh fecal pellets were collected three times at a 2-h interval. All feces collected from each box were pooled as one sample, resulting a total of 3 samples. Meanwhile 3 samples of fresh T1C-19 rice pollen were also obtained. All samples were stored at −80°C. After lyophilization, the concentrations of Cry1C were measured using ELISA as described above.

The total digestion rate of Cry1C in pollen cannot be determined from *Bt* protein concentrations (Cry protein per dry weight) because the digestion process reduces both the amount of Cry protein and the weight of the pollen grains, and the feces also contained mulberry leaf residues. Therefore, the mean Cry protein content of a single pollen grain before ingestion or present in the feces was assessed. The number of pollen grains in 1.0 mg (dw) rice pollen or feces was estimated. Lyophilized fresh pollen or feces (1.0 mg) were mixed with 300 µl fuchsin acid solution. The pollen grains were counted in each of three 5 µl aliquots of the suspension with a microscope at 50× magnification. The mean number of pollen grains in the aliquots was multiplied by 60 to obtain the number in the whole sample. This procedure was repeated seven to 10 times. Based on the mean number of pollen grains in 1.0 mg per fresh rice pollen and feces and the Cry1C protein concentrations in fresh rice pollen and feces, the Cry1C content in single pollen grains was calculated [13].

### Statistical analysis

Student's *t*-tests were conducted to compare Cry1C and Cry2A contents in rice pollen. For the pollen feeding bioassays, one-way ANOVAs followed by Tukey HSD tests were used to determine how the nature of rice pollen (from non-*Bt* rice or from *Bt* rice producing Cry1C and Cry2A) and pollen dose affected pollen consumption, pupation and eclosion rate, and 14-day weight. Because the assumptions for parametric analyses were not met for larval development time (days to pupae), the data were analyzed by Kruskal-Wallis tests, and pair-wise comparisons were further conducted using Mann-Whitney U-tests if significant differences were detected. The Bonferroni correction was applied to correct for 10 pair-wise comparisons leading to an adjusted α = 0.005. At each pollen dose, comparisons were made between each *Bt* pollen treatment (Cry1C and Cry2A) and the control (non-*Bt* pollen treatment) using Dunnett tests. The mean Cry1C concentrations in rice pollen grains before and after gut passage were compared using Student's *t* test.

SPSS 13.0 for Windows was used for all statistical analyses.
